# Drug-related emergency department visits: prevalence and risk factors

**DOI:** 10.1007/s11739-022-02935-9

**Published:** 2022-02-07

**Authors:** Lisbeth Damlien Nymoen, Malin Björk, Trude Eline Flatebø, Merethe Nilsen, Aasmund Godø, Erik Øie, Kirsten Kilvik Viktil

**Affiliations:** 1 Diakonhjemmet Hospital Pharmacy, Oslo, Norway; 2 Department of Pharmacy, University of Oslo, Oslo, Norway; 3 Faculty of Pharmacy, Department of Pharmaceutical Biosciences, Uppsala University, Uppsala, Sweden; 4 Department of Anaesthesia and Intensive Care, Diakonhjemmet Hospital, Oslo, Norway; 5 Department of Internal Medicine, Diakonhjemmet Hospital, Oslo, Norway

**Keywords:** Emergency departments, Medication review, Medication reconciliation, Medication errors, Drug-related hospitalization

## Abstract

The study aimed to investigate the prevalence of drug-related emergency department (ED) visits and associated risk factors. This retrospective cohort study was conducted in the ED, Diakonhjemmet Hospital, Oslo, Norway. From April 2017 to May 2018, 402 patients allocated to the intervention group in a randomized controlled trial were included in this sub-study. During their ED visit, these patients received medication reconciliation and medication review conducted by study pharmacists, in addition to standard care. Retrospectively, an interdisciplinary team assessed the reconciled drug list and identified drug-related issues alongside demographics, final diagnosis, and laboratory tests for all patients to determine whether their ED visit was drug-related. The study population’s median age was 67 years (IQR 27, range 19–96), and patients used a median of 4 regular drugs (IQR 6, range 0–19). In total, 79 (19.7%) patients had a drug-related ED visits, and identified risk factors were increasing age, increasing number of regular drugs and medical referral reason. Adverse effects (72.2%) and non-adherence (16.5%) were the most common causes of drug-related ED visits. Antithrombotic agents were most frequently involved in drug-related ED visits, while immunosuppressants had the highest relative frequency. Only 11.4% of the identified drug-related ED visits were documented by physicians during ED/hospital stay. In the investigated population, 19.7% had a drug-related ED visit, indicating that drug-related ED visits are a major concern. If not recognized and handled, this could be a threat against patient safety. Identified risk factors can be used to identify patients in need of additional attention regarding their drug list during the ED visit.

**Supplementary Information:**

The online version contains supplementary material available at 10.1007/s11739-022-02935-9.

## Introduction

A growing body of evidence suggests that emergency department (ED) physicians do not recognize drug-related ED visits in the fast-paced workflow [1–3]. During the ED visit, physicians evaluate a patient’s symptoms and decide if hospitalization is needed, or if the patient could be discharged directly. If ED visits caused by drug-related issues are not identified during the stay in the ED, physicians might end up misdiagnosing and treating the symptoms instead of the actual problem [1]. Hence, identifying patients with a drug-related referral reason early in the admission process is crucial for the patient safety [1, 4, 5].

The prevalence of drug-related hospital admissions (DRHAs) has been investigated in several studies during the last decades, and the reported prevalence which is summarized in two systematic reviews varies between 1.3 and 41.3% [6, 7]. Recently, there has been a growing interest in investigating drug-related *ED visits* with studies reporting prevalence of 2.3–28.6% [1, 2, 8–11]. Definition of drug-related ED visits, method of identification and population-selection vary between these studies.

The main objective of this study was to investigate the prevalence of drug-related ED visits and risk factors associated with these visits, including involved drug-groups.

## Methods

### Study design

**Fig. 1:**
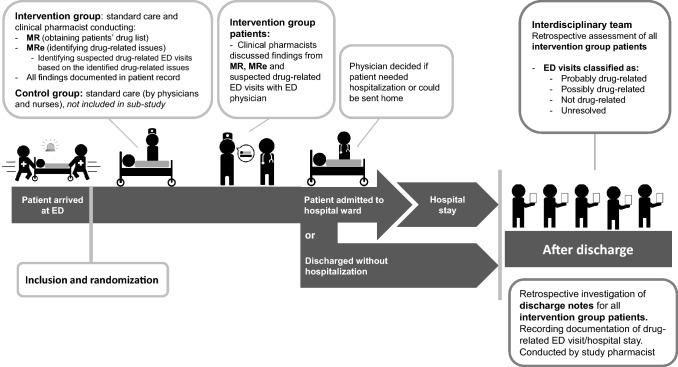
Study design: retrospective cohort study of the intervention group. ED emergency department, MR medication reconciliation, MRe medication review

This retrospective cohort study investigated drug-related ED visits at Diakonhjemmet Hospital, a local urban, non-academic hospital in Oslo, Norway. The study was a sub-study of a randomized controlled trial (RCT), registered at ClinicalTrials.gov, Identifier: NCT03123640, investigating the patients allocated to the intervention group (Fig. 1). Patients were included consecutively in periods from April 2017 to May 2018. A manuscript reporting results from the RCT is in production.

The sub-study was approved by the institutional review board and the Norwegian Regional Committee for Medical and Health Research Ethics and conducted in accordance with the Helsinki Declaration. Written informed consent was obtained from all patients before inclusion. Further, the sub-study was designed and reported according to the STROBE Statement, utilizing the STROBE checklist in all stages of the study (planning, execution, and reporting).

### Study setting

In Norway, patients are referred to a hospital’s ED by health care personnel of the primary health care service e.g., general practitioner (GP), municipal emergency clinic, nursing home physician. GPs and the municipal emergency clinics have a gatekeeper function and handle less severe conditions. Yearly 13,500 patients with both medical and surgical referral reasons are referred to the ED at Diakonhjemmet Hospital. In 2018, the average length of stay in the ED was 3.2 h.

In the RCT, all patients 18 years or older, referred to the ED, and willing to/capable of providing written, informed consent were suitable for inclusion. Patients with both medical and surgical referral reason were included. Unconscious patients were not included e.g., severe intoxications. Further, patients aged ≥  65 with hip fracture were not eligible for inclusion as they were admitted to a specialized ED at another location. Patients were included periodically by study pharmacists between 9:00 am and 10:00 pm, on weekdays and weekends. A total of 807 patients were included in the RCT. Of patients admitted to the ED during data collection periods, 43.7% were assessed for eligibility for inclusion; the remaining patients were not assessed due to ED crowding which exceeded study pharmacists’ capacity. After inclusion, patients were randomized to intervention- or control-group (1:1) with prepacked randomization envelopes from Department of Biostatistics and Epidemiology at Oslo University Hospital.

The intervention group received, in addition to standard care, an intervention consisting of medication reconciliation and systematic medication review conducted by study pharmacists during the patients ED stay (Fig. 1). The intervention was based on the Integrated Medicine Management (IMM) model [12], adjusted to the fast-paced workflow ED-setting [13], and conducted by experienced clinical pharmacists. The medication reconciliation process consisted of a standardized interview of the patient/next of kin/health care personnel to obtain the patient’s complete drug list. Further, written sources (electronical prescriptions, drug list of a multi-dose patient etc.) were checked to clarify and verify information from the interview, and GPs or pharmacies were contacted for complementary information when needed. The medication review was based on the reconciled drug list. In addition, referral notes, examinations in the ED, laboratory test, and computer resources, e.g., interaction databases, summary of product characteristics for drugs, medical databases, were reviewed. Drug-related issues were identified and registered. The study pharmacists documented the reconciled drug list and all clinically relevant drug-related issues, i.e., issues of importance for the patient treatment, including if they suspected the ED visits to be drug-related, in the electronic patient record. These findings were also communicated vocally to the responsible ED physician (Fig. 1). The control group received standard care by physicians and nurses during the ED stay, which did not include systematic medication reconciliation nor medication review.

### Study population

The entire intervention group from the RCT was included in the present sub-study (*n* = 402). Control group patients (*n* = 405) were excluded as systematic medication reconciliation and medication review were not standard care. However, there was no statistical difference in demographics (gender, age, allocation of referral reason, earlier hospital admissions, and hospitalization rate) between the intervention and control groups.

### Data collection

After discharge, a standardized de-identified patient scheme was created for each patient. The schemes included demographic data and results from completed laboratory tests. Further, the schemes included tentative referral reasons set by the referring health care personnel based on the patient’s symptoms and initial examinations (before ED visit). The final diagnoses documented in the discharge note by the physician discharging the patient were also included in the schemes. And finally, the patient’s drug list obtained through medication reconciliation and clinically relevant drug-related issues from the medication review performed in the ED were registered on the schemes (see template in Online Resource 1).

Clinically relevant drug-related issues identified during the medication review were categorized in the following categories: adverse effect: defined as a negative or harmful patient outcome that seemed to be associated with treatment [14]. Non-adherence: defined as deviation between patient’s actual drug use and physician’s prescription with respect to type of drug, dose, or scheme (both unintentional and intentional) [15]. Suboptimal dosing, suboptimal formulation, and need for additional drug treatment: defined as deviation between the patient’s treatment and established national/international guidelines [15]. Inappropriate drug choice: defined as deviation between the patient’s treatment and diagnosis/indication or absolute/relative contraindication [15].

Drugs were classified according to the Anatomical Therapeutic Chemical (ATC)-classification [16, 17]. In the ATC classification system, the active substances are grouped according to the organ or system on which they act (indicated by ATC-1st level), their therapeutic and pharmacological properties (indicated by ATC-2nd–3rd level) and finally their chemical properties (indicated by ATC-4th–5th level). In this study, drugs were reported at ATC classification 3rd level, hereafter called ATC-3 groups.

The patient schemes were presented to an interdisciplinary team (Fig. 1), consisting of two chief physicians and three experienced clinical pharmacists. The interdisciplinary team was blinded regarding if the patient was hospitalized or discharged directly from the ED, they were also blinded to the study pharmacist’s opinion regarding drug-related ED visit. All patient schemes were first assessed and classified by each member of the interdisciplinary team individually. Further, six consensus meetings were arranged between November 2017 and February 2019. The interdisciplinary team classified each ED visit as probably drug-related, possibly drug-related, not drug-related, or unresolved, according to a set of criteria based on World Health Organization Uppsala Monitoring Centre criteria for causality [18] and inspired by Hallas’ criteria for contribution [19]. In the present study, a drug-related ED visit was defined as an ED visit directly (probably) or indirectly (possibly) related to the patient’s drug use prior to the visit. The association between patients’ drug use and ED visits was determined by an interdisciplinary team retrospectively.

To investigate physicians’ recognition of drug-related ED visits/hospital admissions, discharge notes written by physicians treating intervention group patients during ED visit/hospital stay were reviewed by a study pharmacist retrospectively (Fig. 1). It was registered that the treating physician considered the ED visit/hospital admission drug-related if the physician explicitly stated (either through a description or a drug-related diagnosis code) in the discharge note that drugs could be the cause of the visit/admission.

### Statistics

In comparative analysis, probably and possibly drug-related ED visits were treated as one group: drug-related ED visits. Patients not classified (Unresolved/No consensus reached) were not included in the comparative statistics. Data handling was conducted in Microsoft Office Excel 365. Statistical analyses were carried out in Stata SE version 16. Demographic statistics are given as median, interquartile range (IQR), and range for continuous variables and as percentage for categorical variables (group specific percentage when comparing groups). Wilcoxon rank-sum test was used in comparative analysis of continuous variables (due to skewness of data), and Pearson chi^2^-test was used for categorical variables. Logistic regression was used to determine odds ratios of drug-related ED visits, 95% confidence interval (CI). The relative frequency of ATC3 groups was calculated as follows: how often a drug from the specified ATC-3 group was involved in drug-related ED visits divided by number of times drugs from that specific ATC-3 group was used. The tentative referral reasons were presented in text from referring health care personnel and not systematically categorized e.g., with ICD-10 [20]. Hence, the tentative referral reasons were grouped based on the presented text and similar symptoms/tentative diagnoses were grouped together to reduce diversity in data.

## Results

Demographics of the 402 included patients are presented in Table 1. The interdisciplinary team classified 19.7% of the ED visits as drug-related (Table 2). Further, 4.2% of the ED visits were classified as probably drug-related, and 15.4% as possibly drug-related. ED visits could not be classified for 10 of the patients (2.5%), due to lack of necessary information or disagreement within the interdisciplinary team (Table 2).

**Table 1:** Demographics of study population

	Study population *n* = 402
Age	
Median (IQR, range)	67 (27, 19–96)
Patients ≥ 65 years %	54.7
Sex	
Female %	47.8
Male %	52.2
Referral reason allocation	
Medical %	69.7
Surgical %	30.4
Patients admitted to DH last 12 months before ED visit %	31.6
Number of prescribed drugs^a^	
Regular drugs, median (IQR, range)	4 (6, 0–19)
Patients using ≥ 5 regular drugs %	44.3
As needed drugs, median (IQR, range)	2 (3, 0–9)
Responsible for drug administration before ED visit	
Patient %	83.3
Other (next in kin/home care service/ nursing home) %	16.7
Hospitalized patients^b^ %	67.9

*DH* Diakonhjemmet Hospital, *ED* emergency department

^a^Number of prescribed drugs obtained through medication reconciliation

^b^The other part was discharged directly from the ED

**Table 2:** Classification of emergency department (ED) visits by interdisciplinary team

Classification	Number of patients (%) *n* = 402	Sub-classification	Number of patients (%) *n* = 402
Drug-related ED visits	79 (19.7)	Probably drug-related	17 (4.2)
		Possibly drug-related	62 (15.4)
Non-drug-related ED visits	313 (77.9)	−	
Not classified patients	10 (2.5)	Unresolved	4 (1.0)
		No consensus reached	6 (1.5)

Patients classified with a drug-related ED visit by the interdisciplinary team were significantly older and used more drugs regularly compared to patients classified with a non-drug-related ED visit (Table 3). The odds ratio of having a drug-related ED visit was higher in patients with medical referral reasons compared to patients with surgical referral reasons. Patients with a drug-related ED visit were more frequently admitted to hospital after the ED visit (Table 3); this was consistent even after adjusting for age (OR 1.91, 95%CI 1.04, 3.50, *p* = 0.04). Further, referral reasons “hemorrhage or anemia” and “dizziness, syncope, or tendency to fall” were more frequently presented for patients with a drug-related ED visit compared to patients with non-drug-related ED visits.

**Table 3:** Comparisons of demographics

	Drug-related ED visits (*n* = 79)	Non-drug-related ED visits (*n* = 313)	*P* value	OR (95% CI)
Sex				
Female %	54.4	45.7	0.16	1.42 (0.87, 2.33)
Male %	45.6	54.3		
Age				
Age, median (IQR, range)	73 (21, 26–93)	64 (28, 19–96)	< 0.01	1.03(1.01, 1.05)*
Patients ≥ 65 years %	73.4	49.2	< 0.01	2.85 (1.65, 4.92)*
Number of prescribed drugs^a^				
Regular drugs, median (IQR, range)	6 (4, 0–19)	3 (5, 0–15)	< 0.01	1.17 (1.09, 1.25)*
Patients using ≥ 5 regular drugs %	73.4	36.4	< 0.01	4.82 (2.78, 8.35)*
As needed drugs, median (IQR, range)	2 (3, 0–7)	2 (3, 0–9)	0.17	1.11 (0.96, 1.28)
Allocation referral reason				
Medical %	82.3	65.8	< 0.01	2.45 (1.31, 4.56)*
Surgical %	17.7	34.2		
Patients admitted to DH last 12 months %	35.4	30.0	0.35	1.28 (0.76, 2.15)
Responsible for drug administration before ED visit				
Patient %	79.8	84.7	0.29	0.71 (0.38, 1.34)
Other (next in kin/home care service/ nursing home) %	20.3	15.3		
Hospitalized patients %^b^	79.8	64.2	0.01	2.19 (1.21, 3.98)*
Referral reason				
“Hemorrhage or anemia” %	17.7	3.9	< 0.01	5.38 (2.38, 12.18)*
“Malfunction or impaired general condition” %	10.1	4.5	0.05	2.40 (0.97, 5.94)
Dizziness, syncope, or tendency to fall” %	8.9	3.5	0.04	2.66 (1.00, 7.10)*

Patients with drug-related emergency department (ED) visits versus patients with non-drug-related ED visits in the classified study population (*n *= 392)

*DH* Diakonhjemmet Hospital

^a^Number of prescribed drugs obtained through medication reconciliation

^b^The other part was discharged directly from the ED

*Statistically significant (*p* < 0.05)

Adverse effects caused 72.2% of the drug-related ED visits. Further, non-adherence caused 16.5%, and suboptimal dosing caused 7.6% of the drug-related ED visits. Need for additional drug treatment, inappropriate drug choice and suboptimal formulation each caused 1.3% of the drug-related ED visits.

A total of 44 unique ATC-3 groups were found to be involved in drug-related ED visits. Antithrombotic agents were the ATC-3 group most frequently involved in drug-related ED visits (19.0%) (Table 4). Further, antiinflammatory and antirheumatic products, non-steroids (NSAIDs) and agents acting on the renin-angiotensin system (RAS-inhibitors) were each involved in 10.1% of drug-related ED visits. Immunosuppressants, urologicals (drugs for urinary frequency and incontinence) and antidepressants were the ATC-3 groups with the highest relative frequency of drug-related ED visits (Table 4).

**Table 4:** ATC-3 groups involved in drug-related ED visits

ATC-3 group	Relative frequency of drug-related ED visits in ATC-3 groups^a^ %	Proportion of drug-related ED visits caused by specific ATC-3 groups (*n* = 79) %
Immunosuppressants	29.4	3.8
Urologicals (Only drugs for urinary frequency and incontinence were involved in drug-related ED visits)	18.2	5.1
Antidepressants	13.5	3.8
Corticosteroids for systemic use	11.6	6.3
High-ceiling diuretics (loop-diuretics)	10.9	6.3
Antithrombotic agents	10.2	19.0
Drugs for obstructive airway diseases, inhalants (both adrenergics and others)	10.2	7.6
Agents acting on the renin-angiotensin system, with or without thiazide (RAS-inhibitors)	8.2	10.1
Antiinflammatory and antirheumatic products, non-steroids (NSAIDs)	6.7	10.1
Beta blocking agents, with or without thiazide	5.1	6.3

Included ATC-3 groups in the table: either contributed to 5 or more drug-related ED visits, have a relative frequency > 10%, or both. ATC-3 codes of the presented ATC-3 groups can be found at www.whocc.no/atc_ddd_index/

*ATC* Anatomical Therapeutic Chemical classification of drugs

A single drug-related ED visit could involve drugs from multiple ATC-3 groups, and also multiple drugs from the same ATC3-group.

^a^The relative frequency was calculated as follows: how often a drug from the specified ATC-3 group was involved in drug-related ED visits divided by number of times drugs from that specific ATC-3 group were used by the 392 classified patients

Physicians treating the patients during the ED visit/hospital admission had documented a drug-related ED visit/hospital admission in the discharge notes of 11 (2.7%) of the included patients (*n* = 402). Of the 79 ED visits classified by the interdisciplinary team as drug-related, physicians had documented 11.4% in total, 29.4% of all the probably- and 6.5% of all the possibly drug-related ED visits. All discharge notes documenting drug-related ED visits/hospital admissions were written by medical physicians. Surgical physicians did not document any drug-related ED visits/hospital admissions, even though 14 surgical patients (Table 3) were classified with a drug-related ED visits by the interdisciplinary team. The study pharmacists conducting the intervention documented a suspected drug-related ED visit in 82% of the patients classified by the interdisciplinary team to have a drug-related ED visit.

## Discussion

### Prevalence of drug-related ED visits

In this study, 19.7% of the ED visits were classified as drug-related*.* The prevalence of drug-related ED visits/DRHAs in earlier studies varies between 1.3 and 41.3% [1, 2, 6–11]. The prevalence revealed in the present study is however, in line with one prior study investigating drug-related ED visits [2], reporting a prevalence of 22.5%.

One earlier study only identified ED visits caused by adverse drug reactions retrospectively classified based on documentation in electronic patient records and reported a prevalence of 2.3% [9], which is significantly lower than found in the present study. Even though adverse effects were the most frequently registered cause of drug-related ED visits in the present study, other drug-related issues for instance non-adherence and suboptimal dosing accounted for 27.8% of the drug-related visits, similar to prior studies [1, 8]. To estimate the total burden of drug-related ED visits, it is important to focus on more than adverse drug reactions/adverse effects.

Several earlier studies only included hospitalized patients in their population, hence investigating DRHAs [4–7, 21, 22]. In the present study, 20% of the patients classified with a drug-related ED visit were discharged directly from the ED, thus not admitted to hospital. These patients are important to recognize as they also stress the health care service and require adequate evaluation of their drug lists before discharge. Further, some of the DRHA studies only investigated select patient groups, such as patients from specified hospital ward, only patients older than 65 years or using more than five drugs [6, 21, 22]. The difference in patient population between the present study and the above-mentioned DRHA studies makes comparison of prevalence challenging.

National differences in health care systems should also be considered when comparing the prevalence of drug-related ED visits. All patients included in this study were referred to the ED by health care personnel of the primary health care service, which leads to a selected patient population presenting to the ED [23]. A Norwegian study revealed a slightly higher percentage of high-level acute patients (based on triage) presenting to the ED and a higher percentage of patients being hospitalized after the ED stay, compared to EDs in other countries [24]. Further, it was reported that 49.7% of patients admitted to the ED were aged over 65 years [24]. The organizing of the Norwegian health care system could explain why the study population in the present study was older compared to populations of most of the earlier studies investigating drug-related ED visits [1, 8–10], with reported prevalence 2.3–12%. Two earlier studies investigated drug-related ED visits in populations with average age over 60 years and reported prevalence at 22.5–28.6% [2, 11]. This could indicate that the age diversity in the investigated populations may be more important than national differences in health care systems regarding the reported prevalence of drug-related ED visits.

Some of the previous studies investigating drug-related ED visits have used pharmacists to obtain the drug list and reveal drug-related issues [1, 2, 8]. In these studies, the pharmacists classified whether the ED visit was drug-related or not, and independent reviewers were only used when the pharmacist assessments were inconclusive. Prevalence of drug-related ED visits in these studies was reported to be 8.3–22.5%. One earlier study relied on ED physicians’ assessment and documentation in electronic patient records to determine the prevalence of drug-related ED visits, with a reported prevalence of only 3.4% [10]. Another study utilized prospective classification of drug reaction-related ED visits and reported a prevalence of 28.6% [11]. This illustrates the importance of methodology when investigating drug-related ED visits. The present study is the first study combining pharmacist intervention with a retrospective assessment by an interdisciplinary team assessing all patients to determine the prevalence of drug-related ED visits. The combination of prospective intervention and retrospective assessment eliminates several of the limitations of using either of these study designs in an isolated fashion [7, 25]. In addition, utilizing an interdisciplinary team to determine the prevalence, balanced any inter-professional and inter-individual differences of opinion.

### Recognizing drug-related ED visits

To assess drug-related ED visits and DRHAs, it is vital to have a reconciled drug list. To obtain this communication with the patient, next of kin/home care service/nursing home is essential. A study conducted at the same ED as the present study revealed that 62% of the patients had a clinically relevant medication discrepancy between the drug list registered in the hospital’s electronic patient record and the drug list actually in use before visiting the ED [13]. Alongside a reconciled drug list, the interdisciplinary team in the present study was provided essential information from the medication review about non-adherence, suboptimal dosing, and adverse effects, which enabled a thorough assessment of the association between present drug use and the ED visit.

In the present study, only 11.4% of the drug-related ED visits classified by the interdisciplinary team were documented in the discharge notes. This finding is in line with earlier studies raising concerns regarding physicians not recognizing drug-related ED visits/DRHAs [1–3]. Results from this study indicate that physicians are more likely to document an ED visit/hospital stay as drug-related if there is a direct and undoubtedly association (classified as probably) to the patient’s drug use. Only documenting definite drug-related ED visit/hospital admission can lead to neglect of patients who need an adequate evaluation of their drug list. In addition, interpretation of data from the presented study indicates that physicians tend to not document expected events, that is, events that may be interpreted as not preventable in the clinical setting. For instance, none of the infections in patients treated with immunosuppressants were documented to be drug-related by the treating physicians. Patients often depend on follow-up from several different physicians, hence also documenting expected drug-related events are important to alert the next level of care. In addition, documentation can better inform the patient about the risk associated with certain drugs and encourage them to contact health care personnel at an early stage in future.

The preventability of the identified drug-related ED visits was not investigated in the present study. Prior studies have however revealed that between 57.3 and 70.7% of drug-related ED visits may be preventable [5, 8, 10]. According to these studies, ED visits caused by non-compliance, suboptimal dosing, and need for additional drug treatment were most frequently found preventable [8, 10]. Even though all drug-related ED visits may not preventable, it is essential to increase the overall recognition and documentation to be able to avoid the ones that are preventable [8]. Acknowledgment and documentation of suspected/possible drug-related ED visits will increase recognition of drug-related ED visits/hospital admissions.

To identify suspected drug-related ED visits early in the admissions process, this study found that pharmacists can be a valuable resource, which is in line with earlier studies [1, 2]. Additional research is needed to reveal why physicians did not document drug-related ED visits in patients where study pharmacists had documented and communicated their suspicion.

Patients classified with a drug-related ED visit in the present study were more frequently admitted to hospital following their ED stay compared to patients classified with a non-drug-related ED visit. This has also been reported by other studies [2, 8]. A suggested explanation is that identified drug-related issues often require monitored observation to decide on further treatment; hence, hospital admission may therefore be necessary for a greater proportion of patients with drug-related ED visits [8]. In the present study, majority of the drug-related ED visits were not recognized by the treating physicians, which potentially could have delayed the assessment of the patients’ symptoms. The present study revealed that the increased hospitalization rate was not an age-dependent effect; however, there may be some relevant confounding variables which were not controlled for instance triage status and comorbidity. Hence, additional research is needed to determine whether the increased hospitalization rate related to drug-related ED visit patients represents an association, a causation, or both.

### Risk factors for drug-related ED visits

According to the results of the presented study, the risk of having a drug-related ED visit increased with increasing age and increasing number of regular drugs, this is consistent with earlier studies [8, 11]. These risk factors are also in line with the inclusion criteria utilized in some of the prior studies investigating DRHAs [6, 21, 22]. Regarding the aim of the present study, it was, however, essential to include patients without such criteria to identify relevant risk factors. Identifying age as a risk factor also partly explains the higher prevalence revealed in the present study, compared to prior studies with younger populations [1, 8–10]. It is noteworthy that even though a patient aged over 65 years had a significantly higher odds of having a drug-related ED visit, 26.6% of patients classified with a drug-related ED visit were younger than 65 years. And further, 35% of patients classified with a drug-related ED visit used less than five drugs, although patients using more than five drugs had a significantly higher odds of having a drug-related ED visit. This indicates that age and number of regular drugs must be combined with other risk factors to identify all high-risk patients presenting with drug-related ED visits.

In line with the results of this study, medical referral reason was identified as a risk factor for DRHA in one earlier study [26]. The present study did also reveal that none of the identified drug-related ED visits regarding patients with a surgical referral reason were documented by surgical physicians. In earlier studies, surgical referral reasons have been identified to be a risk factor for medication discrepancies [13, 27]. This may indicate that surgeons have more focus on the acute surgical issue rather than reconciling the patients’ drug list, which is essential to reveal drug-related ED visits. Personnel dedicated to conduct medication reconciliation and to identify suspected drug-related ED visits is highly needed in patients with surgical referral reasons.

Immunosuppressants and antidepressants have been identified as risk-drug groups in prior studies [10, 25]. All drug-related ED visits involving immunosuppressants in the present study were infection related (an adverse effect), which is a known complication of the treatment. Infections were however, not found to be more frequent among patients with a drug-related ED visit in this study. Thus, infections were a common referral reason, while relatively few patients used immunosuppressants. Urologicals (drugs for urinary frequency and incontinence) have not been identified as a risk-drug group related to drug-related ED visits/DRHAs in prior studies. However, this group of drugs is capable of causing antimuscarinic side effects, especially in older patients [28]. The antimuscarinic drug burden is increased if combining several antimuscarinic drugs, for instance combination of urologicals and antidepressants [28]. This finding corresponds to identification of “dizziness, syncope, or tendency to fall” as a frequently registered referral reason in patients with a drug-related ED visit. Identifying antithrombotic agents and NSAIDs as risk-drug-groups can correspond to the finding of “hemorrhage or anemia” as the most frequent referral reason for patients classified with a drug-related ED visit. These findings are in line with other studies [2, 3, 25]. RAS inhibitors in older patients can contribute to dizziness and tendency to fall, especially when combined with other blood pressure regulating agents, such as high-ceiling diuretics or beta-blocking agents.

The identified risk factors can be used as screening tools for patients admitted to the ED to prioritize patients in need of a thorough evaluation of their drug list. Older patients with polypharmacy and one of the risk-referral reasons or using drugs from one or more of the risk-drug groups may need extra attention in the ED to assess if the ED visit can be related to their drug use. In addition, the identified risk factors can be used to prevent future drug-related ED visits, as it could alert health care providers in primary health care to perform a systematic medication review to reveal for instance adverse effects, non-adherence, or suboptimal dosage in patients with risk factors.

### Limitations

Given the single study location, in one specific health care system (where patients are referred to the ED by health care personnel of the primary health care system), the results are not necessarily generalizable to other hospital EDs. The revealed prevalence is, however, consistent with other studies investigating study populations with the same age diversity. In addition, most of the identified risk factors are in line with other studies. Indicating that the risk factors may be more generalizable than the prevalence due to the methodology utilized to identify them.

Selection bias cannot be ruled out as only 43.7% of patients admitted to the ED were assessed for eligibility for inclusion to the RCT. However, study pharmacists had no specific criteria for which patients to include in case of ED crowding. And further, summary statistics from 2017 to 2018 in Diakonhjemmet Hospital reveal that 57.2% of patients admitted to the ED were aged over 65 years, hence similar to the age-distribution of the study population in the present study.

A total of 19.7% of the included patients had a drug-related ED visit, indicating that drug-related ED visits are a major concern. The identified risk factors from this study can be used to identify patients in need of extra attention during an ED stay to reveal whether the ED visit is drug-related. Further, the risk factors can also indicate which patients who can benefit from a systematic medication review in the primary health care, which can prevent future drug-related ED visits. Only a minor part of discharge notes written by physicians documented that the ED visit/hospital stay was drug-related, illustrating that this topic needs to be highlighted and an increased awareness regarding possibly drug-related events is needed.

## Supplementary Information

Below is the link to the electronic supplementary material.

Supplementary file1 (PDF 45 kb)

## Data Availability

The data that support the findings are not publicly available due to them containing information that could compromise research participant consent (the patient consent form was approved by the Norwegian Regional Committee for Medical and Health Research Ethics in 2017 and did not include consent to data-sharing). The data are de-identified and stored at the password protected research server at Diakonhjemmet Hospital. Anonymized data are available from the corresponding author on reasonable request.
