# Supplementary material for: Drug-related emergency department visits: prevalence and risk factors
Source: Intern Emerg Med. 2022 Feb 7;17(5):1453–62. doi: 10.1007/s11739-022-02935-9 (PMC9352618; doi:10.1007/s11739-022-02935-9)
Supplement: Supplementary file 1 — Supplementary file1 (PDF 45 kb) [file 11739_2022_2935_MOESM1_ESM.pdf]

Supplementary information -Template standardized patient scheme presented to the interdisciplinary team

Drug-related emergency department visits -prevalence and risk factors, European Journal of Clinical Pharmacology

L. D. Nymoen [1, 2], M. Björk[3], T. E. Flatebø[1], M. Nilsen[1], A. Godø[4], E. Øie[5], K. K. Viktil[1, 2]
[1] Diakonhjemmet Hospital Pharmacy, Oslo, Norway [2] Department of Pharmacy, University of Oslo, Oslo, Norway [3]Department of Pharmaceutical Biosciences, Faculty of Pharmacy, Uppsala University, Uppsala, Sweden [4], Department of Anaesthesia and Intensive Care, Diakonhjemmet Hospital, Oslo, Norway [5] Department of Internal Medicine, Diakonhjemmet Hospital, Oslo, Norway

Corresponding author:
Lisbeth Damlien Nymoen
e-mail: lisbetd@student.matnat.uio.no

Assessment of drug-related emergency department (ED) visits and clinical relevance of drug-related problems[1]

**Patient ID**			
**Gender:**	**Age** (year)**:**	**Department:**	**Triage** (at admission to ED)**:**
**Earlier admitted to Diakonhjemmet Hospital** (yes/no and number of times)**:**			
**Registered adverse drug reactions/CAVE** (drug and reaction)**:**			
**Drug handling before admission:**		**Drug handling problems:**	

**Tentative referral cause** (set by referring physician)	
**Final diagnosis** (from discharge note)	

MEDICAL HISTORY (earlier registered diagnoses)

OTHER RELEVANT INFORMATION

Additional information about the patient was given, for instance:

- Non-adherence
- Social circumstances
- Help from homecare service
- Alcohol abuse

Results from laboratory tests at admission to ED (adjusted according to what was relevant for each patient)

CRP	
Leucocytes	
Neutrophiles	
Lymphocytes	
Hemoglobin	
s-Sodium	
s-Potassium	
s-Calcium	
s-Glucose	
Bilirubin	
Urea	
Creatinine	
GFR	
S-Troponin T	
Pulse	
Blood pressure	

USED REGULAR DRUGS (revealed through medication reconciliation)

**Drug**	**Strength/ dosage**	**Indication**	**Other comments**
Number of rows were adjusted to the findings			

USED AS NEEDED DRUGS (revealed through medication reconciliation)

**Drug**	**Strength/ dosage**	**Indication**	**Other comments**
Number of rows were adjusted to the findings			

[1] The standardized patient scheme was originally in Norwegian, translated for publication

Supplementary information -Template standardized patient scheme presented to the interdisciplinary team

**Drug-related problem (DRP) nr.**	**Description of DRP**	**Actions/ additional comments**
**DRP 1**	Number of rows were adjusted to the findings (all identified DRPs were listed here, regardless of association with a potential drug-related ED-visit)	
**DRP 2**		
**DRP 3**		

<u>ASSESSMENTS (this part were filled out by the interdisciplinary team)</u>

**IS THE ED VISIT DRUG-RELATED? (MARK WITH X):**			
**PROBABLY**	**POSSIBLY**	**NO**	**UNRESOLVED**
**Rationale and suspected drug, associated DRP (free text):**			

**WHEN WAS THE INFORMATION OBTAINED IN MENTIONED DRPs CLINICALLY RELEVANT?**				
	**IN THE ED**	**DURING THE HOSPITAL STAY**	**NOT RELEVANT DURING THE HOSPITAL STAY**	**NOT A DRP**
**LRP 1**				
**LRP 2**				
**LRP 3**				
